# Protection from Yellow Fever by Inoculation

**Published:** 1886-01-20

**Authors:** 


### PROTECTION FROM YELLOW FEVER BY INOCULATION.

All are aware that Dom Pedro, the present Emperor of Brazil, is one of the most scientific savants of the world, and that he has manifested the greatest solicitude for the development of every enterprise looking to the advancement of his Empire. It being brought to his knowledge that some investigations had led to the inference of a special microbial origin of yellow fever, and that preventative inoculation might be resorted to with cultured virus, he authorized the inquiry to be made in a most thorough and scientific method. Compensation for a biologist and microscopist, with a corps of competent assistants, was provided by the Imperial Government, and a series of elaborate experiments was entered upon by Dr. Domingos Friere, with the precautions observed by Pasteur in his investigations. The result of this inquiry verified the anticipation of such a modification of the virus of yellow fever as to admit of its introduction into the human system with only slight and temporary disturbance of the vital organism. The demonstration of the effect of this inoculation in preventing the development of the disease in those who were recognized as being susceptible subjects, has been conducted on a scale which leaves no doubt of its efficacy ; while those exposed under similar conditions have contracted yellow fever, and died from it, none of the five thousand who were inoculated have been seriously attacked during the epidemic. Whatever view may be taken, upon the basis of probable prevention by such a measure, the facts sustain the theory of Pasteur in arresting splenic fever in the inferior creation ; and the observations on so large a number of subjects made by Dr. Friere substantiate the virtue of his process in protecting those inoculated with the attenuated virus from the access of yellow fever.

